# The Impact of Physical Activity on Glycemic Variability Assessed by Continuous Glucose Monitoring in Patients With Type 2 Diabetes Mellitus: A Systematic Review

**DOI:** 10.3389/fendo.2020.00486

**Published:** 2020-07-31

**Authors:** Sebastian L. Bennetsen, Camilla S. Feineis, Grit E. Legaard, Mark P. P. Lyngbæk, Kristian Karstoft, Mathias Ried-Larsen

**Affiliations:** ^1^Centre for Physical Activity Research, Rigshospitalet, University of Copenhagen, Copenhagen, Denmark; ^2^Department of Clinical Pharmacology, Bispebjerg Hospital, Copenhagen, Denmark

**Keywords:** type 2 diabetes, diabetes mellitus, physical activity, exercise, glycemic variability, glycemic control, randomized controlled trials (RCT), continuous glucose monitoring

## Abstract

**Aim:** Patients with Type 2 Diabetes Mellitus (T2DM) have increased risk of developing vascular complications due to chronic hyperglycemia. Glycemic variability (GV) has been suggested to play an even more important role in the risk of developing diabetic complications than sustained hyperglycemia. Physical activity (PA) has shown reducing effects on mean plasma glucose; however, the effect on GV in T2DM needs further description. The objective of this review is to evaluate the effect of PA on GV, assessed by continuous glucose monitoring (CGM) in people with T2DM.

**Methods:** A systematic literature search was conducted on MEDLINE and Embase to find randomized controlled trials (RCTs) covering the aspects T2DM, PA, and CGM. Following eligibility screening, variables of population characteristics, PA interventions, and GV outcomes were extracted and processed through qualitative synthesis. Risk of bias (ROB) was assessed using Cochrane ROB tool v2.0.

**Results:** Of 1,825 identified articles, 40 full texts were screened. In the ten included RCTs matching the eligibility criteria, sample sizes ranged from nine to 63, mean age from 51 (SD 11) to 65 (SD 2) years and mean T2DM duration from four (SD 3) to ten (SD 6) years. Eight RCTs examined GV following single bouts of exercise, while two RCTs examined GV following training interventions. One RCT applied parallel group design, while nine RCTs applied crossover design. Numeric reductions in GV following acute exercise were seen, with four RCTs reaching statistical significance. Numeric reductions in GV were seen following training interventions, with one RCT reaching statistical significance. Numeric reductions of GV after PA appeared independently of intensity and T2DM progression but higher in participants with high baseline HbA1c and GV than with low. 80% of the trials were evaluated as *uncertain/high* ROB.

**Conclusion:** The systematic literature search revealed limited and biased evidence showing that acute PA numerically reduced GV in patients with T2DM. PA reduced GV independently of PA intensity and T2DM progression. Prolonged RCTs with low ROB are needed to confirm reducing effects of PA on GV and to assess the influence of patient- and intervention characteristics on the effect of PA on GV.

## Introduction

Type 2 diabetes mellitus (T2DM) is characterized by increasing insulin resistance concurrent with a not fully compensatory increase in insulin secretion from the pancreatic beta-cells, resulting in pathological hyperglycemia (1). It is well-known that patients with T2DM have increased risk of developing micro- and macrovascular diabetic complications (2).

Increased physical activity (PA) and exercise are cornerstones in the treatment of T2DM (3). Exercise training enhances peripheral and hepatic insulin sensitivity (4) and may improve the function of pancreatic beta-cells in patients with T2DM (5). Moreover, consistent evidence supports beneficial effects of long-term exercise interventions on glycated hemoglobin A1c (HbA1c) in patients with T2DM (6). The measurement of HbA1c reflects the mean plasma glucose level during the last 2–3 months prior to testing and is currently the primary target in the clinical management of hyperglycemia in T2DM (3). It represents a convenient laboratory test, which is not crucially affected by dietary and physical activity behavior immediately prior to testing (7). Increased mean plasma glucose levels are strongly associated with microvascular diabetic complications (2) whereas the association between mean plasma glucose levels and macrovascular complications is less straight forward (8).

However, glycemia is a dynamic process with daily blood glucose fluctuations, varying in amplitude, frequency and duration (9), described as glycemic variability (GV). Glycemic fluctuations, not reflected by HbA1c, may provide additional important information in understanding the risk of developing diabetic vascular complications (10) as increased GV may further increase systemic oxidative stress and inflammation (11, 12). Notably, intraday glycemic variability is associated with macrovascular diabetic complications (13), which are both the leading cause of morbidity and mortality for individuals with T2DM and the largest cost factor in the treatment of the disease (14).

Continuous glucose monitoring (CGM) systems are capable of generating high resolution glucose- profiles, by measuring both diurnal and nocturnal interstitial glucose concentrations in intervals of 5 min. This enables the evaluation of intra-day glycemic control, including GV (15).

While trials support that acute exercise interventions reduce 24-h mean glucose and time spent in hyperglycemia in patients with T2DM (16), little is known about the effect of exercise on GV in patients with T2DM.

The primary objective of this report is to systematically review randomized controlled trials, testing the effect of a PA intervention vs. sedentary/free-living control intervention on GV, assessed by CGM systems in people with T2DM. The secondary objective is to discuss how individual patient characteristics and features of exercise influence the effect of PA on GV in persons with T2DM.

## Methods

This systematic review is reported according to the Preferred Reporting Items for Systematic Reviews and Meta-Analyses (PRISMA) statement (17). A protocol was submitted to the International Prospective Register of Systematic Reviews (PROSPERO) prior to the literature screening process.

### Data Sources and Searches

A systematic literature search was performed including two electronic databases (MEDLINE and Embase). In addition, the reference lists of included articles were examined for additional potential eligible trials. The search string was composed of the following three aspects, based on the primary objective: Type 2 diabetes mellitus, Physical activity, Continuous glucose monitoring profiles. The search string for each aspect was constructed including both Medical/Embase Subject Headings (MeSH/Emtree) and text words. The complete search strings are located in Supplemental Material.

### Eligibility Criteria

Trials matching the eligibility criteria in Table 1 were included.

**Table 1:** Eligibility criteria for the trial selection.

**Inclusion criteria**
Participants with Type 2 diabetes mellitus
Participants (aged ≥ 18)
Interventions with increased PA**^a^** (including exercise)
Sedentary/inactive/free-living control group
CGM reported measures of intra-day glycemic variability**^b^**
Randomized controlled trial
Article written in English language
**Exclusion criteria**
Participants without type 2 diabetes mellitus
Participants with Prediabetes, Impaired glucose tolerance (IGT), Impaired fasting glucose (IFG), Type 1 Diabetes mellitus, Type 1,5 Diabetes mellitus, Gestational diabetes mellitus, Maturity-Onset Diabetes of the young (MODY)
Participants (aged <18)
No physical activity intervention**^a^**
No sedentary/inactive/free-living control group
No available CGM reported measures of intra-day glycemic variability**^b^**
Not a Randomized controlled trial
Article not written in English language

a *PA intervention comprising both structured (exercise) or incidental PA were included, as long as interventions were described by frequency, intensity, type and/or duration*.

*Classification of intensities of the interventions followed American College of Sports Medicine described division of light, moderate and vigorous intensity (18)*.

b *outcome measures of GV should be reported: Standard deviation of plasma glucose (SD_glucose_)/coefficient of variations (CV%)/mean amplitude of glycemic excursions (MAGE)/continuous overall net glycemic action (CONGA-n) or a combination*.

### Study Selection

Identified articles were transferred to Covidence (19) and searched for duplicates. Articles were initially screened by title and abstract and subsequently reviewed full text for inclusion by two independent reviewers (SLB and CSF) according to the eligibility criteria (Table 1). Abstracts containing insufficient information for exclusion by the eligibility criteria were included for full text screening. Conflicts were discussed and resolved by the two reviewers. All included records were finally searched by hand to find additional articles.

### Data Extraction

Data was extracted by one reviewer (SLB) from original articles as well as Supplementary Material. Data of included articles were extracted by five categories:

1. Basic information: authors, trial design.
2. Population characteristics: sample size, sex, age, diabetes duration, HbA1c (mmol/mol), BMI (kg/m^2^), diabetes therapy.
  HbA1c-values stated as (%) was converted to (mmol/mol) for comparability using the online converters from the National Glycohemoglobin Standardization Program (20).
3. Description of the setting and conditions during measurement: CGM system, sampling period, medicine/diet during CGM.
4. Description of the intervention and control intervention: Allocations, description of groups, washout period, Modality, Frequency x Volume, Intensity, Timing. Classification of intensities of the interventions following American College of Sports Medicine described diversion of light, moderate, and vigorous intensity (18).
5. Outcome measures for CGM reported intraday glycemic variability:
  - *SD*_*glucose*_*:* Standard deviation of all glucose readings, measuring the dispersion from mean blood glucose.
  - *CV%:* The ratio of SD to mean glucose, times 100.
  - *MAGE:* Mean amplitude of glucose excursions from peaks to nadirs that are > 1 SD of mean glucose (21).
  - *CONGA-n:* Continuous overall net glycemic action at n- hour(s) (22).

Outcome measures stated as mg/dl was converted to mmol/L using Blood sugar Converter of the Global Diabetes Community (23).

### Risk of Bias Assessment

Risk of bias in individual trials was assessed by two independent reviewers (SLB, CSF). Disagreements were resolved by discussion and clarified with a third reviewer (MRL) if necessary. The Cochrane ROB2.0 tool (2016) for Randomized control trials with cross-over design was used for included trials with cross-over design (24). The Cochrane ROB2.0 tool (2019) for Randomized control trials with parallel-arm design was used for included trials with parallel- arm design (24).

Corresponding authors were contacted by email prior to risk of bias assessment. They were requested to provide eventual protocols, statistical analysis plans or clinical trial registration numbers with predefined outcome lists.

### Synthesis of Results

A scoping literature search prior to this systematic review implied a sparse number and highly heterogeneous trials in the subject field. Meta-analysis was not performed due to this expected large heterogeneity concerning types of interventions, co-interventions and outcome variables throughout the trials. The findings were thus processed through a qualitative synthesis.

## Results

### Study Selection

**Figure 1:**
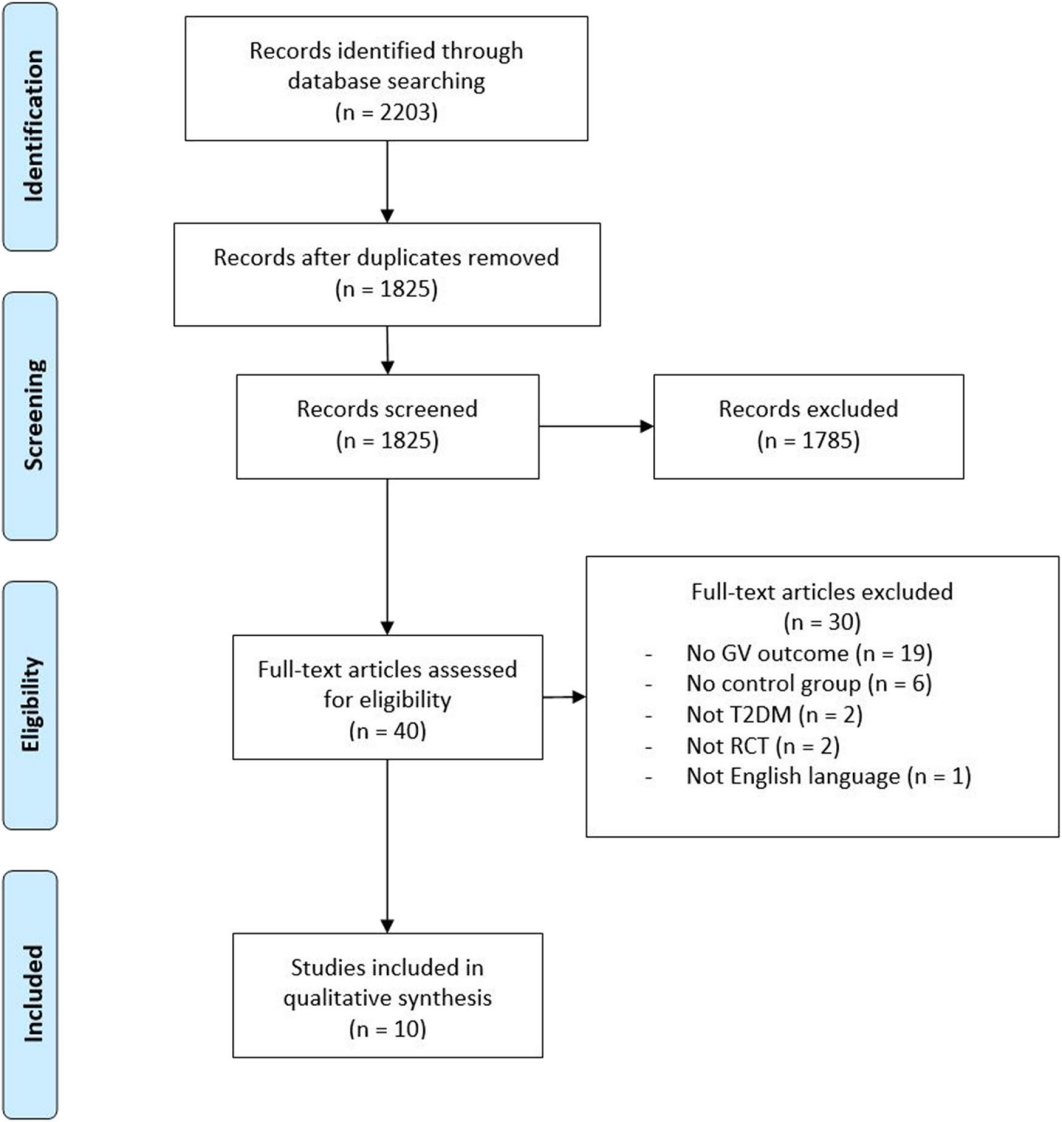
PRISMA flow-diagram visualizing the trial selection.

The initial search on the two online databases from inception to 31st of January 2020 yielded 519 and 1,681 references from MEDLINE and Embase, respectively. With 378 references identified as duplicates, 1,825 articles were left for screening of title and abstract. Following the screening, a total of 40 articles were assessed for eligibility, whereof ten trials were included in this review (25–34). Exclusion of trials was predominantly due to trials not reporting intra-day GV or not having an eligible control group. The flow of the trial selection is shown in Figure 1. Searching the references of included trials did not reveal additional papers.

### Study Characteristics

#### Trial Design

Of the ten included randomized controlled trials, one applied a 3-arm parallel group design (34), while nine applied a crossover design (25–33). Eight of the nine randomized cross-over trials applied single day/bout interventional conditions and reported varying washout periods between conditions ranging from 48 h (32) up to 7 days (25, 26, 28–31, 33), while one trial tested 2 weeks exercise interventions (interval walking training and continuous training) against a control condition (27). Study designs are shown in Table 2.

**Table 2:** Trial characteristics.

**Basic information**		**Population**								**Intervention**						
**Trial (year)**	**Study design**	**Sample size**	**Age**	**Diabetes duration**	**HbA1c**	**BMI**	**Diabetes therapy**	**Habitual PA level**	**Baseline VO2max**	**Allocation**	**Description**	**Washout period**	**Modality**	**Frequency x Volume min**	**Intensity**	**Timing**
		**n (M/F)**	**years (SD)**	**years (SD)**	**mmol/mol (SD)**	**kg/m2 (SD)**	**(n)**		**ml/min/kg (SD)**							
Dempsey (2017)	Crossover	24 (14/10)	62 (6)	6.8 (5.1)	55 (7.7)	33.0 (3.4)	GLM (23), Diet only (1)	<150 min/week of MIE	NR	SIT	Prolonged sitting control	>6 days				
										LW	Sitting interrupted with light-intensity walking or simple resis-tance activities, every 30 min		Walking	12 × 3	3.2 km/h	Pre/post lunch
										SRA			Resistance		Bodyweight	
Haxhi (2015)	Crossover	9 (9/0)	58.2 (6.6)	5.2 (4.3)	53 (6.6)	30.2 (3.1)	GLM (9)	<150 min/week regular PA	NR	CON	Non-exercise control	>1 week				
										SplitEx	Split exercise		Walking	2 × 20	50% HRR	Pre/post lunch
										ContEx	Continuous exercise			1 × 40		Post lunch
Karstoft (2016)	Crossover	14 (11/3)	65 (2)	9 (1)	49 (3.3)	NR	GLM (14)	≤ 90 min/week moderate-intensity PA	23.8 (1.4)^a^	CON	Free-living control	4 weeks				NR
					50 (3.3)				24.6 (1.6)^a^	CWT	Continuous walking at moderate speed	8 weeks	Walking	5 × 60/week for 2 weeks	73% VO2peak	
					49 (3.3)				24.5 (1.5)^a^	IWT	Interval walking: cycles of 3:3 min slow:fast				54/89% VO2peak	
Li and Hu (2018)	Crossover	29 (22/7)	51 (11.2)	5.7 (6)	56.2 (14.5)	24.8 (3.4)	GLM (14), Diet only (15)	≤ 150 min/week regular exercise	NR	CON	Non-exercise control	>7 days				
										MIE	Moderate Intensity Exercise		Walking	1 × 20	40% HRR	Post dinner
Metcalfe (2018)	Crossover	11 (11/0)	52 (6)	4 (3)	52 (9)	28.7 (3.1)	GLM (11)	Classification as moderate active or lower on IPAQ	28.9 (4.8)	CON	Non-exercise control	>5 days				
										REHIT	10 mins unloaded pedaling with 2 × 20 s “all-out” sprints.		Cycling	1 × 10	“All out”	Post breakfast
										HIIT	10 × 60 s 85% Wmax, 60 s low-intensity recovery 25 W			1 × 20	85% Wmax	
										MICT	30 min 50% Wmax continuously effort.			1 × 30	50% Wmax	
Myette-Côté (2015)	Crossover	10 (5/5)	59 (96)	7.7 (5.2)	49 (6.6)	29.5 (4.7)	GLM (10)	NR	NR	CON	Non-exercise(resting)	72 h				
										EX	Exercise		Walking	1 × 50	85% VT	11:00 AM
Rees (2019)	Crossover	63 (29/34)	64 (8)	9.7 (6.1)	51 (8)	30.5 (6.5)	GLM (63)	GLTEQ 35 (± 20)	NR	CON	Seated control	72 h				
										EX	Exercise		Walking	1 × 50	65% HRmax	Pre evening meal
Terada (2016)	Crossover	10 (8/2)	60 (6)	6.8 (4.6)	53.9 (10.9)	30.8 (5.4)	GLM (10)	NR	25.5 (6.6)	CON	Sedentary control	48 h	Walking			
										HIIEfast	Cycles of 1:3 min work:rest			1 × 60	40/100% VO2peak	Pre breakfast
										HIIEfed						Post breakfast
										MICEfast	Moderate intensity continuous exercise				55% VO2peak	Pre breakfast
										MICEfed						Post breakfast
Van (2013)	Crossover	60 (60/0)	60 (6)	8.7 (7.5)	56 (10)	30.1 (3.2)	GLM (34), GLM + INS (17), INS (6), Diet only (3)	NR	NR	CON	Seated control	>7 days				
										EX	Moderate intensity continuous exercise		Cycling	1 × 45–60	50% Wmax	Post breakfast
Winding (2018)	Parallel group	7 (5/2)	57 (7)	7 (5)	53.2 (12.6)	28 (3.5)	GLM (7)	NR	27.2 (9.1)	CON	No training control					NR
		12 (7/5)	58 (8)	6 (4)	52.2 (10.1)	27.4 (3.1)	GLM (11), Diet only (1)		27.8 (5.5)	END	Moderate intensity endurance training		Cycling	3 × 40/week for 11 weeks	50% Wpeak	
		13 (7/6)	54 (6)	8 (4)	51.1 (9.1)	28.1 (3.5)	GLM (13)		28.4 (6.1)	HIIT	Cycles of 1:1 min work:active recovery			3 × 20/week for 11 weeks	95/20% Wpeak	

*BMI, body mass index; GLM, glucose lowering medicine; INS, exogenous insulin therapy; PA, physical activity; MIE, moderate intensity exercise; IPAQ, international physical activity questionnaire; GLTEQ, godin leisure-time exercise questionnaire (± SD); NR, not reported; CON, control condition; SIT, prolonged sitting control; LW, light intensity walking; SRA, simple resistance activities; SplitEx, split exercise; ContEx, continuous exercise; CWT, continuous walking training; IWT, interval walking training; MIE, moderate intensity exercise; REHIT, reduced exertion high intensity training; HIIT, high intensity interval training; MICT, miderate intensity continuous training; EX, exercise; HIIEfast, high intensity interval exercise in fasted state; HIIEfed, high intensity interval training in fed state; MICEfast, moderate intensity continuous exercise in fasted state; MICEfed, moderate intensity continuous exercise in fed state; END, endurance training; HRR, heart rate reserve; VO2peak, maximal oxygen consumption; Wmax, maximal power output; VT, ventilatory threshold*.

a *SEM, standard error of the mean*.

#### Participants

The total number of participants ranged from nine to 63 across the included trials. Three trials included only male participants (26, 29, 33), while the remaining trials included both female and male participants. The age of individuals included in the trials ranged from mean age 51 (SD 11.2) to 65 (SD 2) years (27, 28). The average duration of T2DM in the trial populations ranged from four (SD 3) to ten (SD 6.1) years (29, 31). The investigated populations were categorized as overweight (BMI >25 kg/m2) or obese (BMI >30 kg/m2), with the exception of one trial (28). One paper did not report BMI of included participants (27). Mean HbA1c ranged from 49 mmol/mol (SD 6.6) to 56 mmol/mol (SD 14.5) in the studied populations (30, 33). One trial included a combination of exogenous insulin treated and non-insulin treated participants (33). The remaining trials only included participants without exogenous insulin treatment with varying glucose-lowering drug therapy. One trial partly included drug naïve participants (28). Participants are described in detail in Table 2.

#### Continuous Glucose Monitoring

In general, the included trials calculated GV from interstitial glucose values obtained by a CGM device for periods of between 21.5 and 24 h. One trial calculated GV based on 12 h of CGM (28), while two trials calculated GV pre- and post-intervention, based on glucose monitoring periods of 24 h (27, 34). In all trials, participants did not receive any CGM-training regarding the interpretation of the data. In all trials, CGM systems were calibrated by participants sampling capillary glucose values before main meals and/or bedtime. Diets were standardized with provided meals and snacks during CGM sampling, however one trial instructed the subjects to maintain their habitual diet and keep diet records (34). In that one trial, energy intake was reported similar between monitoring periods, however a significantly higher energy intake was reported in the endurance training group compared to the control group (2,061 kcal/d +/– 694 vs. 1,974 kcal/d +/– 741 *p* < 0.05) in the post-intervention glucose monitoring period. Antidiabetic medicine was continued throughout the glucose monitoring periods of nine trials; however one study withheld glucose-lowering medicine in the morning of experimental days (32). One trial did not explicitly report on the use of medicine during CGM sampling (34). None of the included trials reported changes in antidiabetic medication during the trial period, and the two training intervention trials (27, 34) specifically stated that no changes in glucose-lowering medication was seen in any subjects. The interventional condition was included in the CGM sampling period in seven of the single exercise bout trials, while one trial started CGM sampling directly after the exercise bout (33). Of the two trials monitoring glucose pre and post a training intervention, post CGM was initiated 15–19 (27) and 72 h (34) after the last bout of exercise. Use of CGM in the included trials is summarized in Table 3.

**Table 3:** Continuos glucose monitoring.

**Trial**	**Sensor/System**	**Sampling period**	**Medicine/diet during CGM**
Dempsey (2017)	Enlite/Ipro2, Medtronic	22 h: including intervention	Cont/Std
Haxhi (2015)	NR/iPRO, Medtronic	24 h: including intervention	Cont/Std
Karstoft (2016)	Enlite/Ipro2, Medtronic	24 h: starting 15–19 h post last exercise bout	Cont/Std
Li and Hu (2018)	NR, Medtronic	12 h: including intervention	Cont/Std
Metcalfe (2018)	Enlite/Ipro2, Medtronic	24 h: including intervention	Cont/Std
Myette-Côté (2015)	NR/Ipro2, Medtronic	21, 5 h: including intervention	Cont/Std
Rees (2019)	Enlite/Ipro2, Medtronic	24 h: including intervention	Cont/Std
Terada (2016)	NR/Ipro2, Medtronic	24 h: including intervention	Cont, witheld in the morning/Std
Van Dijk (2013)	GlucoDay S, A. Menarini Diagnostics	24 h: starting post conditions	Cont/Std
Winding (2018)	Sof/Guardian Real-Time, Medtronic	48 h: starting 72 h post last exercise	NR/DR

*CGM, continuous glucose monitoring; h, hours; Cont, medicine continued during CGM; Std, standardized diet during CGM; DR, diet records; NR, not reported*.

#### Interventions

Eight of the included trials assessed the effect of PA on GV following a single session of exercise or increased PA by interrupted sitting. Two trials evaluated the effect of training interventions of two (five sessions/week) and 11 (three sessions/week) weeks, respectively (27, 34). Five of the included trials tested continuous exercise conditions (26, 28, 30, 31, 33). Four trials tested both interval and continuous exercise conditions (27, 29, 32, 34). One trial tested interrupted sitting with light intensity walking or simple resistance exercises (25). Continuous exercise interventions were described as moderate intensities, with one trial testing a vigorous intensity intervention, aiming for 73% VO_2peak_ (27). Bouts of interval exercise consisted of alternating vigorous intensity intervals and rest periods (27, 29, 32, 34). The volume of the exercise bouts ranged from ten to 60 min. Interventions are described in detail in Table 2.

#### Comparators

All eight included acute exercise (single session) trials had an inactive control intervention through either a seated control intervention (25, 31, 33) or a not further described no-exercise control intervention (26, 28–30, 32). The two included training intervention trials had either a free-living control intervention (27) or a not further described no training control intervention (34). Control interventions are shown in Table 2.

#### Outcomes

In general, reported outcome measures of GV differed across the included trials. Five trials reported one outcome measure for GV (27, 30–32, 34), two trials reported two outcome measures (29, 33), two trials reported three outcome measures (26, 28) and one trial reported four outcome measures for GV (25). Mean amplitude of glucose excursions (MAGE) was the most frequently used outcome measure of GV and reported in eight trials. SD_glucose_, CV% and CONGA was reported in four, three and four trials, respectively. Results of the individual trials are summarized in detail in Table 4.

**Table 4:** Changes in outcome measures of GV in the individual trials.

**Trial**	**Outcome measure**	**Group**	**Results**	**Between group difference**	
**(Sample)**			**Mean (SD)**	**Δmean**	**Δ%**
Dempsey et al. (2017) (*n =* 24)	Sdglucose	SIT	2.3 (0.1)^a^		
		LW	1.9^*^ (0.1)^a^	−0.4	−17.4
		SRA	1.8^*^ (0.1)^a^	−0.5	−21.7
	CV%	SIT	19.8(1.2)^a^		
		LW	21.8 (1.2)^a^	2	10.1
		SRA	20.7 (1.2)^a^	0.9	4.5
	MAGE	SIT	5.9 (0.3)^a^		
		LW	4.6^*^ (0.3)^a^	−1.3	−22
		SRA	4.3^*^ (0.3)^a^	−1.6	−27.1
	CONGA-1	SIT	2.0 (0.1)^a^		
		LW	1.6^*^ (0.1)^a^	−0.4	−20
		SRA	1.5^*^ (0.1)^a^	−0.5	−25
Haxhi et al. (2015) (*n =* 9)	Sdglucose	CON	1.5 (0.5)		
		SplitEx	1.3 (1.0)	−0.2	−13.3
		ContEx	1.5 (0.6)	0	0
	MAGE	CON	3.4 (1.4)		
		SplitEx	3.3 (2.4)	−0.1	−2.9
		ContEx	3.4 (1.1)	0	0
	CONGA-1	CON	6.6 (1.0)		
		SplitEx	6.4 (1.5)	−0.2	−3.0
		ContEx	6.5 (0.9)	−0.1	−1.5
	CONGA-2	CON	6.1 (1.0)		
		SplitEx	5.9 (1.3)	−0.2	−3.3
		ContEx	6.1 (0.9)	0	0
	CONGA-4	CON	5.6 (1.0)		
		SplitEx	5.6 (0.9)	0	0
		ContEx	5.6 (0.9)	0	0
Li and Hu et al. (2018) (*n =* 29)	SDglucose	CON	1.2 (0.5)		
		MIE	1.0^*^ (0.4)	−0.2	−16.7
	CV%	CON	15.6 (7.1)		
		MIE	12.8^*^ (6.4)	−2.8	−17.9
	MAGE	CON	3.1 (1.6)		
		MIE	2.6 (1.9)	−0.5	−16.1
Metcalfe et al. (2018) (*n =* 11)	MAGE	CON	4.21 (2.04)		
		REHIT	3.76 (1.35)	−0.45	−10.7
		MICT	3.47 (1.59)	−0.74	−17.6
		HIIT	3.42 (1.50)	−0.79	−18.8
	CONGA	CON	7.25 (1.00)		
		REHIT	6.69 (0.72)	−0.56	−7.7
		MICT	6.93 (0.93)	−0.32	−4.4
		HIIT	6.89 (0.53)	−0.36	−5
Myette-Côté et al. 2015 (*n =* 10)	MAGE	CON	3.4 (1.5)		
		EX	3.9 (1.7)	0.5	14.7
Rees et al. (2019) (*n =* 63)	MAGE		Period 1:		
		CON	4.5 (2.2)		
		EX	4.2 (1.8)	−0.3	−6.7
			Period 2:		
		CON	4.1 (1.8)		
		EX	3.7 (1.4)	−0.4	−9.8
Terada et al. (2016) (*n =* 10)	MAGE	CON	5.03 (2.10)		
		HIIEfast	−1.79^†^ (−3.18 to −0.41)^b^		−35.6
		HIIEfed	−0.26 (−1.70 to 1.18)^b^		−5.2
		MICEfast	−1.54^*^ (−2.98 to −0.10)^b^		−30.6
		MICEfed	−0.98 (−2.42 to 0.46)^b^		−19.5
Van Dijk et al. (2013) (*n =* 60)	Sdglucose	CON EX	Over the 24 h after exercise there was a tendency observed for a reduction in SDglucose (*P =* 0.06)		
	CONGA	CON EX	Over the 24 h after exercise, a significant reduction was observed for CONGA1, CONGA2, and CONGA4 (*P < * 0.05)		
**Trial**	**Outcome measure**	**Group**	**Sample**	**Pre**	**Post**	**Within group effect**	
			**(n)**	**Mean (SD)**	**Mean (SD)**	**Δmean**	**Δ%**
Karstoft et al. (2016)	MAGE	CON	14	5.2 (0.4)^a^	6.4 (0.6)^a^	1.2	23.1
		CWT	14	6.5 (0.7)^a^	6.5 (0.7)^a^	0	0
		IWT	14	7.1 (0.6)^a^	5.4 (0.4)^†^ ^a^	−1.7	−23.9
Winding et al. (2018)	CV%	CON	7	22 (7)	20 (7)	−2	−9.1
		END	12	24 (10)	21 (9)	−3	−12.5
		HIIT	13	22 (7)	17 (4)	−5	−22.7

a *standard error of the mean (SEM)*.

b *changes from control (95% Confidence Interval). Sdglucose, MAGE and CONGA: mmol/L. Between group differences equals interventional condition vs. non-exercise control*.

*CON, non-exercise control; SIT, prolonged sitting; LW, light intensity walking; SRA, simple resistance activities; SplitEx, split exercise before and after meal; ContEx, continuous exercise after meal; MIE, moderate intensity exercise; HIIT, high intensity interval training; REHIT, reduced-exertion high intensity interval training; MICT, moderate-vigoruos-intensity continuous training; HIIEfast, high-intensity interval exercise fasted-state; HIIEfed, high-intensity interval exercise post-breakfast; MICEfast, moderate-intensity continuous exercise fasted-state; MICEfed, moderate-intensity continuous exercise post-breakfast; CWT, continuous walking training; IWT, interval walking training; END, endurance training*.

* *statistically significant reduction from control group (p < 0.05)*.

† *statistically significant reduction from control group (p ≤ 0.01)*.

### Glycemic Variability

#### Effects of Acute Exercise (Single Bout)

Eight trials with a total of 216 participants provided data on GV following a single bout of exercise. Overall, three of the trials reported significant reductions in GV comparing exercise to control (25, 28, 32), whereas no trials reported significant increases in GV comparing exercise to control. Four trials with a total of 122 participants reported SD_glucose_ as outcome measure for GV (25, 26, 28, 33). Between-intervention differences in mean SD_glucose_ comparing exercise to control group ranged from −21.7% (−0.5 mmol/l) to 0% (0 mmol/l). One trial did not report SD_glucose_ quantitatively but reported a reduction in SD_glucose_ (*p* = 0.06) (33). Two trials with a total of 53 participants reported CV% as outcome of GV (25, 28). Between intervention differences in mean CV% comparing exercise to control group ranged from −17.9% (−2.8%-points) to +10.1% (+2.0%-points). Seven trials with a total of 156 participants reported MAGE as outcome of GV (25, 26, 28–32). Between intervention differences in mean MAGE, comparing exercise to control group ranged from −35.6% (−1.79 mmol/l) to +14.7% (+0.5 mmol/l) (30, 32). Four trials with a total of 104 participants reported CONGA as outcome measure for GV (25, 26, 29, 33). Between intervention differences comparing exercise to control group ranged from −25.0% (−0.5 mmol/l) to −1.5% (−0.1 mmol/l) for CONGA 1 (25, 26). Between intervention difference in CONGA 2 ranged from −3.3% (−0.2 mmol//l) to 0% (0 mmol/l). No quantitative changes in CONGA 4 were reported (26). One trial did not report CONGA quantitatively, but reported a significant reduction in CONGA-1, −2, and −4 (*p* < 0.05) (33).

#### Effects of Training Interventions (Weeks)

Two trials provided data on GV following 2 and 11 weeks of exercise, respectively (27, 34). Overall, training interventions reduced GV numerically compared to control, however only one of the trials reported significant reductions in GV comparing a training intervention to control. Following ten bouts (2 weeks) of interval or continuous walking, interval walking significantly reduced MAGE compared to the control group (*p* = 0.01), while the continuous walking did not. MAGE decreased with −23.9% (−1.7 mmol/l, *p* = 0.02 vs. baseline) in the interval intervention and 0% (0 mmol/l, *p* > 0.05 vs. baseline) in the continuous walking intervention, while MAGE increased with +23.1% (1.2 mmol/L, *p* > 0.05 vs. baseline) in the control intervention (27). Following 33 bouts (11 weeks) of interval or continuous cycling, no significant differences between the intervention groups and the control groups were reported for GV. CV% decreased with −22.7% (−5%-points, *p* < 0.05 vs. baseline) within the interval group and with −12.5% (−3%-points, *p* > 0.05 vs. baseline) within the continuous group, while within-group change in the control group was −9.1% (−2%-points, *p* > 0.05) (34).

#### Effects of Different Intensities

Overall, numeric reductions in GV following PA and exercise were present across the intensities of interventions, and significant reductions were reported following both light-, moderate-, and high intensity interventions. One trial evaluated GV following sitting interrupted by 3 min of light intensity PA of either walking (LW) or simple resistance activities (SRA). Interrupting sitting with both light intensity modalities (LW and SRA) significantly reduced SD, MAGE, CONGA1 compared to prolonged sitting. Interrupting sitting with both SRA and LW increased GV measured by CV, compared to prolonged sitting (25). Table 4 shows results in detail.

Seven trials evaluated the acute effect of continuous moderate intensity exercise on GV, compared to a control intervention. Three trials reported between intervention changes from control in SD_glucose_ ranging from −16.7% (−0.2 mmol/l) to 0% (0 mmol/l) (26, 28, 33). Six trials reported between intervention changes in mean MAGE ranging from −30.6% (−1.54 mmol/l) to 14.7% (+0.5 mmol/l) (26, 28–32). One trial reported a between intervention change from control in CV% of −17.9% (−2.8%-points, *p* = 0.009), following a single bout of continuous moderate intensity walking (28). One trial reported a non-significant within-group difference in CV% of −12.5% (−3%-points), following 11 weeks of continuous moderate intensity cycling, compared to a within-group difference of −9.1% (−2%-points) in the control group (34).

Four trials investigated GV following a high intensity interval intervention (27, 29, 32, 34). Two trials reported between intervention changes from control in MAGE of −35.6% (−1.79 mmol/l) to −5.2% (−0.26 mmol/l) after an acute bout of high intensity exercise (29, 32). Another trial reported a within intervention change in MAGE of −23.9% (−1.7 mmol/l, *p* = 0.02 vs. baseline) after 2 weeks (ten sessions) of interval walking, significantly reducing GV compared to control group (*p* = 0.01) (27). One trial reported a within group difference in CV% of −22.7% (−5%-points, *p* < 0.05) after 11 weeks of high intensity interval (HIIT) cycling, compared to a within-group difference in CV% of −9.1% (−2%-points, *p* > 0.05) in the control group (34).

#### Timing of Exercise

One trial evaluated the acute effect of exercise on GV before- and after breakfast, reporting significant reductions in GV when exercise was performed in a fasted state (*p* = 0.015 fasted vs. fed). Between intervention differences in MAGE, comparing fasted high intensity interval exercise (HIIEfast) and fasted moderate intensity continuous exercise (MICEfast) to the control group was −35.6% (−1.79 mmol/l, *p* < 0.01) and −30.6% (−1.54 mmol/l, *p* < 0.05), respectively. Exercising in fed state reduced MAGE from control with −5.2% (−0.26 mmol/l, *p* > 0.05) and −19.5% (−0.98 mmol/l, *p* > 0.05) for HIIEfed and MICEfed, respectively (32).

#### Diabetes Disease Progression

Both diabetes duration and the need for exogenous insulin (i.e., reflective of the loss of beta-cell function) can be indicators of progression of T2DM. Comparable numeric reductions in GV following acute exercise was reported across indicators of diabetes progression.

One trial included both exogenous insulin and non-insulin treated patients with T2DM. Reductions in GV (SD_glucose_ and CONGA-1, −2, −4) in the period of 24 h after 45–60 min of continuous moderate intensity exercise, was reported comparable regardless of exogenous insulin treatment. However, there were reported higher levels of GV (CONGA-4 and SD_glucose_, *p* < 0.05) in the exogenous insulin-treated patients than in the non–insulin-treated patients. In addition, diabetes duration [12 (SD 7.7) vs. 6.6 years (SD 6.6)] and HbA1c [60 mmol/mol (SD 11) vs. 54 mmol/mol (SD 9)] was higher in the exogenous insulin-treated patients than in the non-insulin treated patients (*p* < 0.05) (33).

The trial including patients with shortest mean diabetes duration entailed patients with mean diabetes duration of four (SD 3) years. In this population, reported between intervention differences after an acute bout of either sprint-, high intensity interval- or moderate intensity continuous exercise ranged from −18.8% (−0.79 mmol/l) to −10.7% (−0.45 mmol/l) in MAGE, and from −7.7% (−0.56 mmol/l) to −4.4% (−0.32 mmol/l) in CONGA, when comparing to the control group (29). The trial including patients with longest mean diabetes duration entailed patients with mean diabetes duration of 9.7 (SD 6.1) years. In this population, reported between-intervention differences in MAGE after an acute bout of continuous walking ranged from −9.8% (−0.4 mmol/l) to −6.7% (−0.3 mmol/l), when comparing to the control group (*p* = 0.137) (31).

#### Effects of Glycemic Control

Overall, numeric reductions of GV after acute exercise were greater in trials with participants with higher HbA1c and GV at baseline. The single exercise bout trial including participants with the lowest mean HbA1c [49 mmol/mol (SD 6.6)], reported between-intervention difference in MAGE after an acute bout of continuous walking of +14.7% (+0.5 mmol/l), when compared to control group (*p* = 0.37) (30). Two trials included participants with mean HbA1c of 55 mmol/mol (SD 7.7) and 56.2 mmol/mol (SD 14.5) reporting between-intervention difference in MAGE after an acute bout of continuous walking or 8 h period interrupting sitting with walking ranging from −22% (−1.3 mmol/l) to −16.1% (−0.5 mmol/l) (25, 28). In trials comparing exercise with a control group with a low GV (mean MAGE ≤ 3.4 mmol/l) the effects of an acute exercise bout ranged from −16.1% (−0.5 mmol/l) to +14.7% (+0.5 mmol/l) (26, 28, 30). In trials comparing exercise with a control group with a high GV (mean MAGE ≥ 4.1 mmol/l) the effects of an acute exercise bout ranged from −35.6% (−1.79 mmol/l) to −6.7% (−0.3 mmol/l) (25, 29, 31, 32).

### Risk of Bias

**Figure 2:**
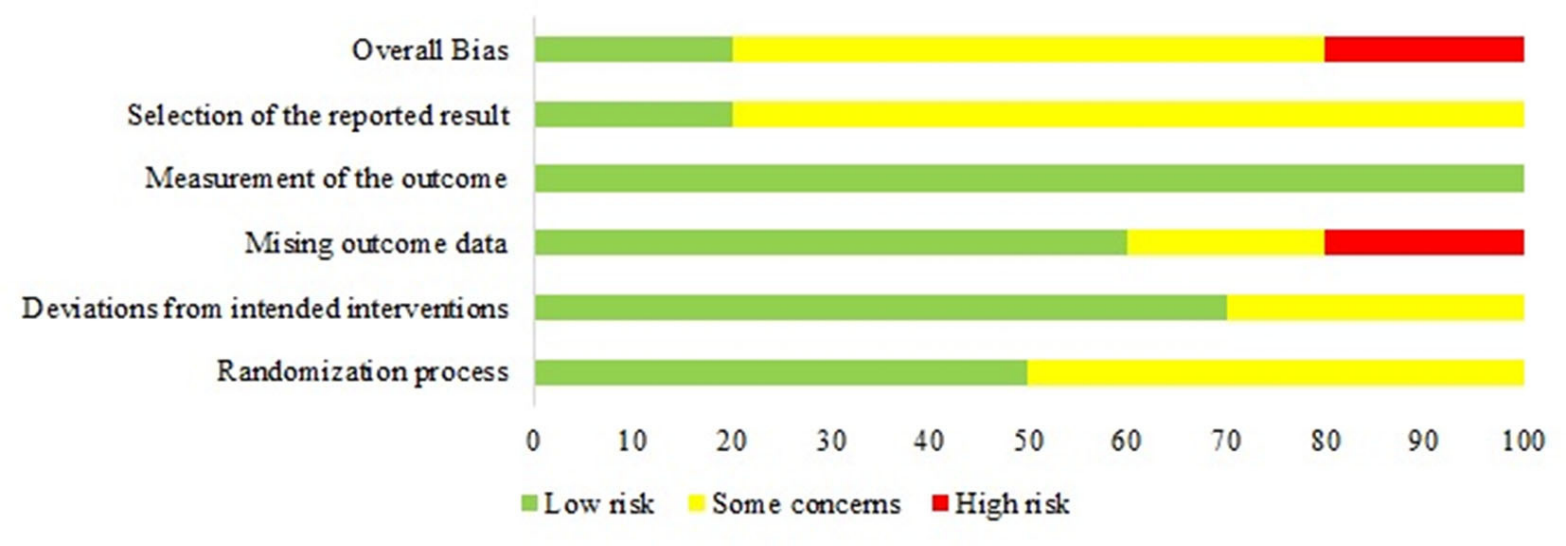
Visualizing risk of bias as percentage in each domain.

Overall, 20% of the trials were rated as *low risk* of bias, while 60% were rated *some concerns* and 20% were rated as *high risk* of bias. Figure 2 visualizes the risk of bias as percentage across the domains.

Half of the trials insufficiently described the randomization and in particular the concealment process, and were thus evaluated as *some concerns* in the Randomization process Domain (26, 28, 30, 32, 33).

Ascribed to the nature of the PA interventions, all participants and personnel were aware of the intervention. No crucial deviations from the intended interventions were reported in seven of the ten trials. However, two trials were evaluated as *some concerns*, due to insufficient description of deviations from the intervention (33) or missing information about a washout-period (30).

In the missing data domain, two trials were evaluated as *some concerns*, due to insufficient description of missing data (26, 33). Two trials were evaluated as *high risk*, because the missing data was unequally divided across interventions and due to insufficient sensitivity analysis handling the missing data (31, 32).

In the domain about the measurement of the outcome, all trials were evaluated as *low risk* due to the assumption that CGM systems were not affected by missing assessor blinding.

**Figure 3:**
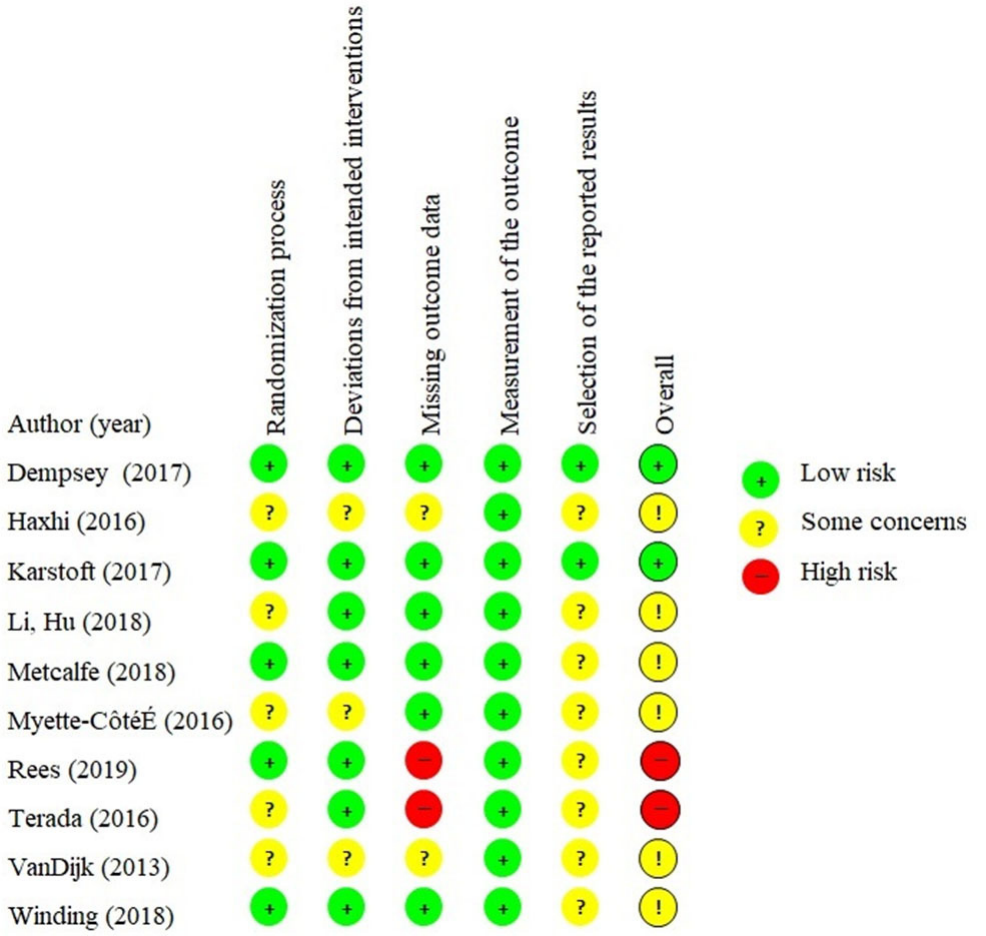
Visualizing risk of bias of individual domains across included trials.

Eight trials could not accommodate our inquiry of a sufficiently described and predefined list of outcomes in terms of a protocol, statistical analysis plan, or comprehensive clinical trial registration and were thus evaluated as *some concerns* in the domain about selective reporting of the results. Two trials were evaluated as *low risk*, due to either transparent reporting of all available outcomes for GV, despite the absence of a predefined outcome list (25) or a predefined outcome list (27). Figure 3 visualizes the risk of bias in individual domains across the included trials.

## Discussion

### Summary of Evidence

The literature search revealed that a limited number of ten trials have been conducted to examine the effect of exercise on GV in persons with T2DM. Notably, only two studies reported effects of prolonged training interventions (≥2 weeks duration). Across acute exercise trials, only three trials reached statistical significance, however a general tendency of exercise reducing GV was reported when assessed by absolute measures (SD_glucose_, MAGE, CONGA), though the effect attenuated when adjusted for mean glucose (CV%). The reductions in GV in response to exercise appeared present across intensities, as well as comparable for patients varying in progression of T2DM. There was a signal toward PA reducing GV more in trials including participants with either high HbA1c or high GV than in trials including participants with lower HbA1c or GV. Finally, 80% of the included trials were associated with uncertain (60%) or high-risk (20%) of bias.

In absolute measures, intra-day GV appears to decrease in response to exercise. With MAGE as the most frequently reported absolute GV measure, the reductions could be related to minimizing the amplitude of hyperglycemic excursions following meals (21). With the insulin-independent glucose uptake in skeletal muscle increasing acutely by muscle contractions (35), an exercise bout could attenuate the amplitude and duration of the post-prandial hyperglycemia when performed in continuation of a meal (36). Several of the included acute exercise trials, applied an exercise bout following breakfast or other main meals, why the reductions of GV could be driven by the attenuation of the ongoing post-prandial hyperglycemia at the time of the exercise bout. Furthermore, the enhanced insulin-dependent glucose uptake is shown to be present 24 h after a single bout of exercise in T2DM patients (37). Thus, the decrease in absolute GV could also be related to attenuation of meal-related hyperglycemia for following meals during the day of exercise. On the other hand, one trial indicated that exercising in a fasted state pre-breakfast could be advantageous in order to decrease MAGE (32). By exercising in a fasted state, the substrate availability of the exercising muscle is limited, which could lead to a greater glycogen depletion of the skeletal muscle. While an inverse relationship between glycogen content and insulin-stimulated glucose uptake has been demonstrated (38), it can be speculated that the glycogen depletion following fasted state exercise is greater than when exercising fed (39), leading to increased insulin-dependent glucose uptake beneficial for reducing the GV for the following time period.

The international consensus on the use of CGM of the American Diabetes Association recommends CV% as the primary outcome measure for GV (9). By adjusting for the mean glucose concentration, the response to PA conditions compared to control in one acute trial led to increases in GV (CV%), which contrasted with simultaneously reporting substantial reductions in absolute measured GV and 24-h mean glucose (25). When reporting changes in GV relative to the mean glucose concentration, the effect of exercise appeared to diminish. The interpretation might be that as the relative magnitude of GV is similar to the control conditions, the glucose fluctuates around lower means, possibly with reduced absolute amplitude of the excursions. Accordingly, no significant difference in CV% was reported between the intervention groups and the control group following an 11-week exercise intervention (34). However, the missing effect of the long-term exercise intervention on GV could also be due to missing standardization of the diet during CGM measurement and low power of the trial. Energy intake after the intervention was reported significantly lower in the control group compared to the endurance exercise group, potentially diminishing a possible reducing effect of exercise on GV (34).

In the reviewed literature, the decrease in the intra-day GV in response to a single bout of exercise seems to be present following light-, moderate-, and vigorous intensity exercise. However, regarding the evaluation of the effect of exercise intensity, the trials of Karstoft and Terada could be illuminated, as the design of the energy-matched interventions makes direct comparison of intensity possible (27, 32). In the trial of Karstoft et al. (27) 2 weeks of interval walking training reduced MAGE significantly compared to control; while energy-expenditure matched continuous walking training did not change GV. This potentially indicates that intensity might play a role in order to minimize GV. Well in line with previous suggestions of the intensity of the exercise being closer related to improving glycemic control than the volume of exercise (40). In contrast, the trial of Terada et al. (32) showed that a bout of high intensity interval exercise reduced MAGE equivalent to an energy-matched bout of moderate intensity continuous exercise. Further trials using energy-matched exercise interventions are needed to assess the influence of exercise intensity on GV in the T2DM population.

An interesting, though speculative finding of the reviewed literature was that the decrease in GV in response to acute exercise was present across the spectrum of diabetes duration as well as in patients using exogenous insulin treatment. With T2DM progressing over time, by increasing insulin resistance and impaired beta-cell function, the need of antidiabetic drugs to maintain glycemic control increases, and eventually, the beta-cell function is impaired to an extent where exogenous insulin is needed. It is proposed, that the ability to recover the beta-cell function by exercise is dependent of the remaining insulin secretory capacity (5) and that the capability to regain the insulin response to ingested glucose is vital in order to achieve remission (41). This advocates that the effect of exercise primarily benefits patients with shorter diabetes duration not yet in need of exogenous administered insulin. Nevertheless, regular exercise has previously been shown to be beneficial for T2DM patients using exogenous insulin, by reducing doses of exogenous insulin needed to maintain glycemic control (42). This suggests that the ability to improve insulin sensitivity in response to exercise is preserved in T2DM patients, despite an impaired beta-cell function and longer diabetes duration, well in line with the indications of this review. However, due to the short duration of the majority of the trials, the differential effects of exercise according to remaining beta-cell function or diabetes duration may not appear. If the mechanisms of improved beta-cell function involve beta-cell rest due to an increased peripheral insulin sensitivity and thus reduced glucotoxicity or decreased low-grade inflammation (43), the trial duration should be prolonged (>4 weeks) until such effects would become apparent (44). Literature suggests that long-term adaptations from regular exercise improve glucose metabolism through increased glucose uptake capacity but may decrease the temporary insulin sensitizing effect after an acute bout of exercise (45). Although speculative, baseline physical activity and/or fitness may therefore modify the response of acute exercise on GV, with a potentially attenuated effect in the more active/fit participants. However, the heterogeneous and relatively poor standard of reporting baseline PA habits and physical fitness throughout most of the included trials makes an interpretation of the effect of physical activity habits at baseline and physical fitness at baseline on the effect of physical activity on GV challenging.

### Limitations

Trials matching the eligibility criteria were limited, of small sample sizes and with GV as a secondary outcome of CGM-measurements. Hence, the available trials were neither designed nor powered to address GV as an outcome, which could influence on the results of GV in either direction. Additionally, the lack of consensus on how to report GV implies difficulties in both summarizing the effect of PA on GV and comparing the effects across interventions and population characteristics. The heterogeneity in the reported GV outcomes and interventions across the included trials limits this work in addressing the effect of modality, sex, ethnicity, and other prognostic factors of T2DM on exercise induced changes in GV.

The missing consensus on GV outcome measures and the fact that most of the included trials were completed without predefined outcome measures for GV increases the risk of bias in selective outcome reporting. Additionally, a substantial number of articles was excluded at full text level of the trial selection for not reporting GV but reporting other measures of high-resolution glucose profiles. The fact that GV is not reported, though being a simple computable outcome when CGM data are available could imply a publication bias. Another interpretation of this finding, though beyond the scope of this review, could be that the clinical importance of GV as a risk factor in diabetic vascular complications is continuously debated (46, 47). Overall, the risk of bias assessment implies a general weakness in the evidence of the included trials arising mainly from non-transparent reporting of outcomes and insufficient descriptions of processes like concealment and handling of missing data.

The literature search of this systematic review did not include evidence from unpublished trials and gray literature which limits the ability of the present work to account for possible publication bias. Additionally, only articles written in English language were included in the work, which could induce a selection bias of the present systematic review.

The standardization of “free-living” between the intervention and control groups during CGM monitoring is crucial for the accurate assessment of the effect of the intervention on GV. Included trials principally standardized diet and glucose lowering medicine during the CGM-monitoring periods, and some also accounted for eventual compensatory PA during CGM-periods using accelerometers. However, varieties in behavioral factors besides diet and PA like e.g., sleep patterns, as well as use of common painkillers like acetaminophen (48) and other still unknown factors could influence the CGM profiles and thereby induce confounding of the results of individual trials and thereby to this review. Furthermore, the CGM profiles could be influenced by the use of different sensors throughout the studies. Although speculative, the different accuracies of the sensors (49) could potentially influence the comparisons of the results of the different trials. Moreover, the interventional conditions of the acute PA trials were predominantly performed during the CGM monitoring period. It is shown that plasma glucose concentration increases in persons with T2DM during vigorous exercise (37), why the intervention itself could induce glycemic excursions, possibly reducing the effect of exercise when comparing especially higher intensity exercise with a non-exercise control group. Along those lines, the reporting standard of baseline PA patterns and baseline physical fitness is relatively poor or absent throughout most of the studies. This potentially affects the effect of PA on GV.

## Conclusion and Implications for Research and Practice

The systematic reviewed literature revealed a limited body of evidence examining the effect of PA on CGM derived measures of GV in patients with T2DM, especially for prolonged engagement in exercise interventions. Acute exercise seem to reduce GV, yet only few trials reached statistical significance. The beneficial reducing effect of acute exercise on GV was attenuated when adjusting for mean glucose. Moreover, the non-significant reducing effect of exercise on GV appeared present independently of exercise intensity and progression of T2DM. However, there was a signal of additional beneficial effects of higher intensity and/or interval exercise. Further, there was a signal toward PA reducing GV more in trials including participants with either high HbA1c or high GV than in trials including participants with lower HbA1c or GV. The heterogeneity of interventions regarding PA intensity, modality, volume, and outcome measurements across the trials makes the assessment of the effect of prognostic factors like PA modality, sex, age and others on GV difficult. Finally, the findings should be interpreted warily due to the uncertain ROB across the trials. Future research with GV as predefined primary outcome is needed to determine if exercise reduces GV significantly and if this reduction is independent of reductions in mean glucose. In order to reduce the risk of bias in future trials, a protocol with a predefined outcome list should be available. It would be of great interest to assess the effects of long-term exercise as well as different intensities and modalities of exercise on GV. Moreover, the assessment of the effect of different prognostic factors such as varying progression of T2DM, sex, age, and others on GV, and on the effect of PA on GV would be of great clinical relevance.

## Data Availability Statement

The original contributions presented in the study are included in the article/Supplementary Material, further inquiries can be directed to the corresponding author/s.

## Author Contributions

SB, ML, and MR-L initially designed the project with inputs from KK. CF and SB searched and reviewed the literature and assessed risk of bias of included trials in consultation with MR-L and wrote the manuscript with inputs and critical feedback from GL, ML, KK, and MR-L. SB extracted data. SB and CF contributed equally to the work. All authors accepted the final manuscript.

### Conflict of Interest

MR-L received a speakers fee from Novo Nordisk A/S. The remaining authors declare that the research was conducted in the absence of any commercial or financial relationships that could be construed as a potential conflict of interest.
