# Supplementary material for: The Impact of Physical Activity on Glycemic Variability Assessed by Continuous Glucose Monitoring in Patients With Type 2 Diabetes Mellitus: A Systematic Review
Source: Front Endocrinol (Lausanne). 2020 Jul 31;11:486. doi: 10.3389/fendo.2020.00486 (PMC7438766; doi:10.3389/fendo.2020.00486)
Supplement: Supplementary file 1 [file Data_Sheet_1.docx]

**Supplementary material**

**Search strategy**

|  | **Population** | **Continuous Glucose monitoring** | **Physical activity** |
| --- | --- | --- | --- |
| **MesH** | Diabetes mellitus, type 2 | Blood Glucose Self-Monitoring | Exercise  Exercise therapy |
| **Emtree** | Non insulin dependent diabetes mellitus/ | Blood glucose monitoring/ | Exp physical activity/  Exp exercise/ |
| **Text Word / .ab.kw.ti.** | Type 2 Diabetes Type II diabetes Diabetes mellitus type 2 Diabetes mellitus type II Diabetes type 2 Diabetes type II T2DM T2D DMT2 Non insulin dependent diabetes Noninsulin dependent diabetes NIDDM | Continuous glucose AND (sens* OR profil*)  Glucose monitor*  CGM  CGMS  Medtronic AND (enlite OR guardian OR IPro2 OR minimed OR paradigm)  Abbott AND (freestyle OR libre OR glucose monitor OR navigator)  Dexcom AND (G4 OR G5 OR G6 OR Seven)  GlucoMen OR GlucoDay | Physical activit*  Exercise  Training  Running  Jogging  Cycling  Bicycling  Swimming  Walking  Ergometer  Treadmill  Stair climbing  Physical conditioning  Endurance  Physical fitness  HIIT |

**Search string for MEDLINE**

((((("Diabetes Mellitus, Type 2"[Mesh]) OR ((((((((((((((((((((((Type 2 diabetes[Text Word]) OR Type II diabetes[Text Word])) OR Diabetes mellitus type 2[Text Word])) OR Diabetes mellitus type II[Text Word])) OR Diabetes type 2[Text Word])) OR Diabetes type II[Text Word])) OR T2DM[Text Word])) OR T2D[Text Word])) OR DMT2[Text Word])) OR Non insulin dependent diabetes[Text Word])) OR Noninsulin dependent diabetes[Text Word])) OR NIDDM[Text Word]))) AND (("Blood Glucose Self-Monitoring"[Mesh]) OR ((((((((((continuous glucose[Text Word]) AND ((((profil*[Text Word]) OR sensing[Text Word]) OR sensor*[Text Word]) OR monitor*[Text Word]))) OR glucose monitoring[Text Word]) OR glucose monitor[Text Word]) OR glucose monitors[Text Word]) OR ((CGM[Text Word]) OR CGMs[Text Word])) OR ((Medtronic[Text Word]) AND (((((enlite[Text Word]) OR guardian[Text Word]) OR Ipro2[Text Word]) OR minimed[Text Word] OR paradigm[Text Word])))) OR ((Abbott[Text Word]) AND ((((freestyle[Text Word]) OR libre[Text Word]) OR glucose monitor[Text Word]) OR navigator[Text Word]))) OR ((Dexcom[Text Word]) AND ((((G4[Text Word]) OR G5[Text Word]) OR G6[Text Word]) OR Seven[Text Word])))) OR GlucoMen[Text Word] OR GlucoDay[Text Word]) AND (((("Exercise"[Mesh]) OR "Exercise Therapy"[Mesh])) OR ((((((((((((((((Exercise[Text Word]) OR training[Text Word]) OR physical activit*[Text Word]) OR running[Text Word]) OR jogging[Text Word]) OR cycling[Text Word]) OR swimming[Text Word]) OR walking[Text Word]) OR ergometer[Text Word]) OR treadmill[Text Word]) OR stair climbing[Text Word]) OR physical conditioning[Text Word]) OR endurance[Text Word]) OR physical fitness[Text Word] OR bicycling[text word]))))

**Search string for EMBASE**

|  | Search |
| --- | --- |
| Text word (.ab.kw.ti) | 1. (Type 2 Diabetes or Type II diabetes or Diabetes mellitus type 2 or Diabetes mellitus type II or Diabetes type 2 or Diabetes type II or T2DM or T2D or DMT2 or Non insulin dependent diabetes or Noninsulin dependent diabetes or NIDDM).ab,kw,ti.  2. ((Continuous glucose and (sens* or profil*)) or Glucose monitor* or CGM or CGMS or (Medtronic and (enlite or guardian or IPro2 or minimed or paradigm)) or (Abbott and (freestyle or libre or glucose monitor or navigator)) or (Dexcom and (G4 or G5 or G6 or Seven)) or (GlucoMen or GlucoDay)).ab,kw,ti.  3. (Physical activit* or Exercise or Training or Running or Jogging or Cycling or Bicycling or Swimming or Walking or Ergometer or Treadmill or Stair climbing or Physical conditioning or Endurance or Physical fitness or HIIT).ab,kw,ti.  4. Non insulin dependent diabetes mellitus/  5. Blood glucose monitoring/  6. Exp physical activity/ OR Exp exercise/  7. 1 OR 4  8. 2 OR 5  9. 3 OR 6  10. 7 AND 8 AND 9 |
| Emtree terms |  |
| Combined |  |
